# Genetic engineering in diatoms: advances and prospects

**DOI:** 10.1111/tpj.70102

**Published:** 2025-03-16

**Authors:** Yixuan Li, Longji Deng, Emma Jane Lougheed Walker, Bogumil J. Karas, Thomas Mock

**Affiliations:** ^1^ School of Environmental Sciences University of East Anglia Norwich Research Park Norwich NR4 7TJ UK; ^2^ Department of Biochemistry, Schulich School of Medicine and Dentistry Western University London Ontario N6A 5C1 Canada

**Keywords:** diatoms, genetic engineering, metabolic engineering, synthetic biology

## Abstract

Diatoms are among the most diverse and ecologically significant groups of photosynthetic microalgae, contributing over 20% of global primary productivity. Their ecological significance, unique biology, and genetic tractability make them ideal targets for genetic and genomic engineering and metabolic reprogramming. Over the past few decades, numerous genetic methods have been developed and applied to these organisms to better understand the function of individual genes and how they underpin diatom metabolism. Additionally, the ability of diatoms to synthesize diverse high‐value metabolites and elaborate mineral structures offers significant potential for applications in biotechnology, including the synthesis of novel pharmaceuticals, nutraceuticals, and biomaterials. This review discusses the latest developments in diatom genetic engineering and provides prospects not only to promote the use of diatoms in diverse fields of biotechnology but also to deepen our understanding of their role in natural ecosystems.

## Significance Statement

Diatoms are not only responsible for a significant proportion of global annual primary production, but they are also the most diverse group of algae. Our review discusses the latest developments in diatom genetic engineering and provides prospects not only to promote the use of diatoms in diverse fields of biotechnology, but also to deepen our understanding of their role in natural ecosystems.

## INTRODUCTION

Diatoms are a diverse class of photosynthetic microalgae widely distributed across oceans, lakes, rivers, and even soils (Pinseel et al., 2020). Representing the most diverse phytoplankton class in today's oceans, diatoms are estimated to include approximately 100 000 species worldwide (Mann & Vanormelingen, 2013). With only about 0.1% of the total global plant biomass, diatoms contribute over 20% of the world's primary productivity (Bar‐On et al., 2018; Leblanc et al., 2012; Malviya et al., 2016). This disproportionate impact underscores their critical role, especially in aquatic ecosystems, driving global biogeochemical cycles.

Given their ecological significance and diversity, there is an impetus to sequence and develop molecular tools for a variety of diatom species. The current availability of over 50 diatom genomes with >100 still to be sequenced as part of the 100 Diatom Genomes Project (100DGP) in addition to more than 500 diatom transcriptomes, provides a significant resource for studying diatom biology from genes to applications (Brodie et al., 2017). Based on these resources, researchers have gained important insights into diatom physiology, evolution, and their ecological functions. Since the first successful genetic transformation in *Cyclotella cryptica* and *Navicula saprophila* using biolistics, this method has rapidly become a standard and, therefore, is the most popular technique for the genetic modification of diatoms (Dunahay et al., 1995). Advances in transformation technologies, such as electroporation (Hu & Pan, 2020; Okada et al., 2023; Yin & Hu, 2021; Zhang & Hu, 2014) and conjugation (Karas et al., 2015), combined with the advent of Clustered Regularly Interspaced Short Palindromic Repeats (CRISPR/Cas9) (Hopes et al., 2016; Nymark et al., 2016), have enabled precise and efficient editing of diatom genomes. These advancements have positioned diatoms as an attractive target for genetic engineering and metabolic reprogramming, facilitating applications in biotechnology, such as alternative fuels, pharmaceuticals, nutraceuticals, and the synthesis of novel biomaterials (Brodie et al., 2017). This review aims at providing an overview of the methods and applications involved in the genetic manipulation of diatoms, including the current progress in terms of further developing genomics resources as the foundation of genome engineering. Our review will also provide prospects for this fast‐developing field of fundamental and applied research with diatoms.

## DIATOM GENOMIC RESOURCES

*Thalassiosira pseudonana* and *Phaeodactylum tricornutum* were the first diatoms to have their genome completely sequenced (Armbrust et al., 2004; Bowler et al., 2008). Characterized by small genome size (<35 Mb) and rapid growth (>one cell division per day under optimal conditions), both species quickly became models, providing key insights into diatom evolution, adaptation and ecological roles in their natural environments (Bowler et al., 2010; Mock et al., 2022). However, the availability of only two diatom genomes is insufficient to understand the diversity of diatoms (Nakov et al., 2018). To gain more comprehensive insights on diatom adaptation and evolution, additional diatom species have been subsequently sequenced. For example, the genome of *Fragilariopsis cylindrus* revealed how diatoms adapt to polar oceans (Mock et al., 2017) and the genome of *Nitzschia putrida* illuminated how diatoms evolve from a phototropic to a heterotrophic lifestyle (Kamikawa et al., 2022). Furthermore, the genome of oleaginous diatoms *Fistulifera solaris* and *C. cryptica* provide a foundation for improving lipid synthesis through genetic modifications (Tanaka et al., 2015; Traller et al., 2016), which is relevant for diverse biotechnological applications. However, a step‐change in our understanding of diatom functional and evolutionary genomics will be provided by the ‘100 Diatom Genomes Project’ (https://jgi.doe.gov/csp‐2021‐100‐diatom‐genomes/), which was initiated a few years ago by an international consortium of diatom researchers from different fields. The project is funded by the Joint Genome Institute (JGI), Department of Energy (USA).

Consequently, these fast‐expanding diatom genomic resources will provide a wealth of novel biological information which can be accessed through public data banks such as the National Center for Biotechnology Information (NCBI), the Joint Genome Institute (JGI), the European Nucleotide Archive (ENA), the Ensembl Genome Browser, DiatOmicBase, and PLAZA (Table S1). However, to harness this wealth of information, it will be necessary to develop synthetic biology tools that allow for large‐scale genome engineering, analogous to the methods that have been pioneered for prokaryotes (Fredens et al., 2019; Hutchison et al., 2016) and yeast (Schindler et al., 2024). In combination with multi‐omics tools, the integration of these diverse resources and methods will provide holistic insights into the function of genes, genetic networks, and their role in diverse biological processes.

## GENETIC ENGINEERING

There are two complementary approaches used so far for diatom genetic engineering: Forward and reverse genetics. The former is a phenotype‐first approach, which starts with an observable phenotype as the outcome of random mutagenesis. The latter aims to purposefully modify a genetic locus of interest, resulting in novel phenotypes to be characterized to reveal the *in vivo* function of the genetic locus. Reverse genetics is an important component of synthetic biology to create new diatom biology, which in its most developed stage would generate a synthetic diatom based on artificially synthesized chromosomes that carry a novel combination of genes and genetic networks. Although there is no synthetic diatom yet, significant advancements in terms of large‐scale chromosome‐like assemblies and novel transformation protocols pave the way for achieving that goal.

### Forward genetics

Forward genetics generates random mutations by either chemical and/or radiation mutagenesis. DNA mutations can also be caused by random integrations of exogenous DNA, including selective markers (Goold et al., 2024; Moosburner et al., 2022). High‐throughput approaches are subsequently used to screen many of the treated cell lines to identify phenotypes that are significantly different from the wild‐type (WT) controls. For instance, chemically mediated mutagenesis in diatoms using *N*‐ethyl‐*N*‐nitrosourea (ENU) has identified a new type of uridine‐5‐monophosphate synthase (UMPS) (Sakaguchi et al., 2011) in *P. tricornutum*. UV‐mediated mutagenesis has been used to introduce dominant genetic mutations in diatoms. For example, it has been successfully applied to increase the eicosapentaenoic acid (EPA) content in *P. tricornutum* by up to 44% (Alonso et al., 1996). Additionally, UV mutagenesis was applied to identify novel selectable markers, such as the genetic locus conferring resistance against the herbicide norflurazon, which was caused by a specific amino acid substitution in the phytoene desaturase (PDS) gene (Taparia et al., 2019). Thus, forward genetics is a valuable tool for introducing dominant mutations. However, the diploid nature of diatom genomes during most of the life cycle imposes challenges to obtaining biallelic (homozygous) gene inactivation by untargeted mutagenesis, especially if the mutagen primarily causes single‐strand breaks in the DNA (Huang & Daboussi, 2017; Moosburner et al., 2022). Similar challenges exist for the application of reverse genetics approaches.

### Reverse genetics

**Figure 1:**
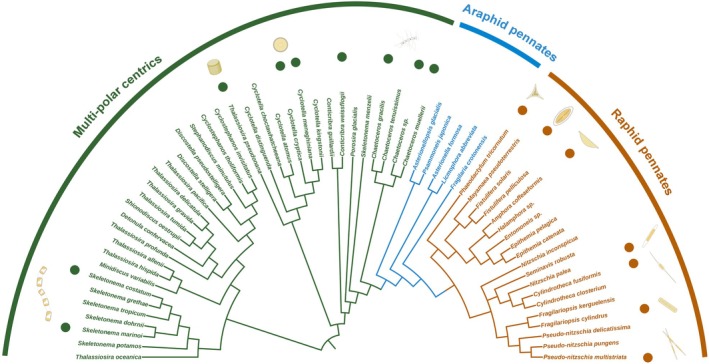
Maximum‐likelihood phylogenetic tree and transformation status of sequenced diatom genomes based on the 18S sequences. The phylogenetic tree depicts the evolutionary relationships among diatom species with complete genome sequences. Species with established genetic transformation protocols are highlighted with dots at the leaves. Representative morphologies of key species are illustrated alongside their respective phylogenetic positions. The tree is divided into three major groups based on the phylogeny: multi‐polar centrics (green), araphid pennates (blue), and raphid pennates (brown).

Before any genetic loci can be modified using targeted approaches, there need to be methods for delivering and establishing genetic material in the diatom cell. Usually, these transformation protocols are developed using the expression of marker genes such as green fluorescent protein (GFP) and yellow fluorescent protein (YFP), which, based on their fluorescence, help to assess the stability of the genetic modification. The latter is an important criterion for many applications in reverse genetics with diatoms (Faktorova et al., 2020; Falciatore et al., 1999; Sabatino et al., 2015; Yin & Hu, 2021). Once a transformation protocol has been developed, many endogenous genetic loci can be manipulated. The most used genetic modifications in diatoms include gene overexpression, knockdown, and knockout. Furthermore, some diatoms such as *T. pseudonana* can be used for efficient gene replacement facilitated by homology‐directed repair (HDR). Until now, the genetic transformation systems have been established in 20 diatom species (Figure 1).

Gene overexpression, defined as an unnaturally high level of gene expression usually caused by the knock‐in of an additional copy of the target gene driven by a strong constitutive promoter such as fucoxanthin chlorophyll‐a/c‐binding protein (*FCP*), may give cells new phenotypic characteristics, allowing researchers to draw conclusions about the potential function of the overexpressed gene. This method was successfully employed for the first time in diatoms in 1995 (Dunahay et al., 1995).

Gene knockdown is the process by which gene expression is at least reduced and sometimes completely abolished. It is reversible as the method targets mRNA instead of DNA, thereby resulting in the decrease of the encoded protein. The first report of gene knockdown was based on inhibiting the expression of the GUS reporter and the endogenous cryptochrome gene in *P. tricornutum* using antisense RNA technology (De Riso et al., 2009). Since then, gene silencing has been widely applied in diatoms (Sabatino et al., 2022; Shrestha & Hildebrand, 2017; Trentacoste et al., 2013; Wang et al., 2023). However, the molecular mechanism underlying gene silencing remains to be completely resolved because it does not appear to be following the canonical mechanism of RNA interference (RNAi) mediated by small non‐coding RNAs (sRNAs) and the activity of the endoribonuclease Dicer (DCR) because the mRNA of the silenced genes is still present, as observed in *T. pseudonana*, for instance, (Kirkham et al., 2017). However, new data based on comparative genome and transcriptome analyses and reverse genetics with P. *tricornutum* have in fact revealed that key RNAi effectors have diversified in diatoms. Nevertheless, this new study suggests the presence of distinct RNAi pathways in diatoms. For instance, the *P. tricornutum* DCR was found to process 26–31‐nt‐long double‐stranded sRNAs originating mostly from transposons covered by repression‐associated epigenetic marks (Grypioti et al., 2024).

In addition to gene overexpression and silencing in diatoms, the last decade has revolutionized our ability to modify the diatom genome by enabling targeted modifications using sequence‐specific nucleases. Modifications are achieved through the application of meganucleases (MNs), transcriptional activator‐like effector nucleases (TALEN) and especially CRISPR/Cas‐assisted genome editing. These genetic tools introduce double‐strand DNA breaks (DSB) at specific loci, with subsequent repair either via homology‐directed repair (HDR) or non‐homologous end joining (NHEJ). The latter is the most frequent repair mechanism in diatoms acting on single‐nucleotide mutations and small insertion–deletions (INDELS). MNs, TALENs, and CRISPR/Cas have been used successfully to induce targeted mutations in diatom genomes (Daboussi et al., 2014; Fortunato et al., 2016; Hopes et al., 2016). Among these three gene modification methods, meganucleases are the oldest method which recognizes and cleaves long DNA target sites (14–40 bp) and tolerates minor sequence changes in these sites (Chevalier & Stoddard, 2001). TALENs consist of a sequence‐specific DNA‐binding domain and a FokI cleavage domain (Christian et al., 2010). The customized TALE DNA‐binding domain guides the nuclease to the target gene, while the FokI nuclease domain dimerizes to induce DNA cleavage (Bogdanove & Voytas, 2011; Voytas, 2013). The first proof of concept for using MNs and TALENs to edit diatom genomes was published by Daboussi et al. (2014). TALEN technology was applied to both *P. tricornutum* and *T. pseudonana* (Fortunato et al., 2016; Weyman et al., 2015). TALENs have the advantage of low off‐target activity, ensuring high specificity in genome editing (Nemudryi et al., 2014). However, a typical TALEN system requires pairing two TALENs to target DNA sequences of 30–36 base pairs, with each side consisting of 18 repeats (Malzahn et al., 2017). Thus, the design is complex and not as versatile as the CRISPR/Cas technology. Therefore, MNs and TALENs were gradually replaced in diatom research by the effective CRISPR/Cas technology (e.g., Hopes et al., 2016). The native CRISPR‐Cas9 system functions as part of the bacterial immune system by detecting and cutting foreign DNA using sequence‐specific guide RNAs (gRNAs). In genome editing, the CRISPR RNA (crRNA) fused to the trans‐activating CRISPR RNA (tracrRNA) generates single‐guide RNAs (sgRNAs), which replace native gRNAs. The sgRNAs guide the Cas9 protein to cleave the DNA at the desired loci (Bortesi & Fischer, 2015). The first reports for successful CRISPR/Cas‐based genome editing leading to stable gene knockouts were based on work with *P. tricornutum* (Nymark et al., 2016) and *T. pseudonana* (Hopes et al., 2016). Both papers came out in the same year, separated only by some weeks. Following these landmark papers, CRISPR/Cas‐mediated homologous recombination (HR) was developed in *P. tricornutum*, *T. pseudonana*, and *Chaetoceros muelleri*, providing targeted genome editing at endogenous loci (Belshaw et al., 2022; Moosburner et al., 2020; Yin & Hu, 2023). In 2018, the first paper on DNA‐free gene knockout by direct delivery of CRISPR‐Cas9 ribonucleoproteins (RNPs) was published. This DNA‐free method is still successfully used in the diatom community (Serif et al., 2018). Furthermore, a Cas9 nickase (nCas9) has recently been developed for diatom research. The native Cas9 nuclease has two active domains, that is, RuvC (D10A) and HNH (H840A). The RuvC domain is inactive in the Cas9 nickase (nCas9). Thus, nCas9 only introduces single‐strand DNA breaks (nicks). The nCas9 system has recently been used in diatoms to suppress the off‐target effects of the native Cas9 nuclease (Matsui et al., 2024; Nawaly et al., 2020; Nigishi et al., 2024).

Very recently, a dead (d)Cas9 has been used in diatom research for the first time (Guo et al., 2024). The dCas9 is characterized by its inability to cut DNA. However, it still retains the ability to bind to DNA (Pickar‐Oliver & Gersbach, 2019). dCas9 in diatoms has been used for CRISPR interference (CRISPRi) to perform CRISPR‐mediated knockdown in *P. tricornutum* (Guo et al., 2024). dCas9 can also be used for CRISPR activation, in which the dCas9 is fused with transcriptional activators, allowing the upregulation of target genes. However, this method (CRISPRa) has yet to be established for diatom research. Base editors (e.g., cytosine, adenosine) have not yet found their way into diatom research either (Lee et al., 2023). Base editors enable precise editing of single bases, which can be used to introduce single amino acid changes to confer, for instance, resistance to compounds such as cycloheximide and norflurazon (Stevens et al., 2001; Taparia et al., 2019).

## MOLECULAR GENETIC TOOLKITS

### Promoters

Promoters can be endogenous or heterologous in origin and can drive constitutive or inducible expression of the respective downstream open reading frame. Commonly used promoters for diatom research are listed in Table 1. Constitutive promoters facilitate stable gene expression and are not influenced by growth stage or environmental conditions. Examples of endogenous constitutive promoters commonly used in diatoms include those from genes encoding fucoxanthin chlorophyll‐a/c‐binding protein (*Fcp*, now called *Lhcf*), histone 4 (*H4*), elongation factor II (*ef2*), acetyl‐CoA carboxylase (ACCase) and U6 small nuclear RNA (U6) (Apt et al., 1996; De Riso et al., 2009; Dunahay et al., 1995; Falciatore et al., 1999; Hopes et al., 2016; Nymark et al., 2016; Sabatino et al., 2015; Seo et al., 2015; Siaut et al., 2007). Recently, *in vivo* transcriptional activity of four new endogenous promoters from the NADH:ubiquinone oxidoreductase (*Nub*), Synaptobrevin/VAMP‐like protein (*SVP*), predicted protein 45,582, nd Prohobitin (*Pbt*) was characterized in *P. tricornutum* along with additional alternative promoters (e.g., calmodulin‐dependent protein kinase II, oxygen‐evolving enhancer protein 3, glucose‐6‐phosphate isomerase, fructose bisphosphate aldolase), providing comparative data for advancing genetic engineering strategies (Garza et al., 2023; Windhagauer et al., 2021). However, it should be mentioned that not all endogenous constitutive promoters are equally strong. For example, *H4* appears to be a strong promoter, whereas *FCP* is considered weaker in *P. tricornutum* (Garza et al., 2023).

**Table 1:** Toolbox for diatom transformation

	Species	Delivery system	Selective marker	Reporter	Promoter	Approach	Cloning and assembly strategy	Reference
Raphic pennate	*Phaeodactylum tricornutum*	Biolistics Electroporation Conjugation PEG	*Sh ble*, *nat*, *bsr*, *sat*, *nptII*, *cat*	eGFP, LUC, GFP, GUS, YFP, CFP, VENUS, mCherry	*FCP*, *H4*, *EF2*, *U6*, *Nub*, *SVP*, *45582*, *Pbt*, *Act2*, *GLNA*, *calm*, *oee3*, *flav*, *NR*, *Isi1*, *FBP1*, *ca1*, *AP1*, *CMV*, *CIP*, *CaMV*, *RSV‐LTR*, *DIG/pUAS*, *XVE/OlexA*	Overexpression, TALENs, MNs Gene silencing, CRISPR	Restriction enzyme Golden Gate Gibson uLoop *In vivo*	Cochrane, Brumwell, Soltysiak, et al. (2020); Diamond et al. (2023); Falciatore et al. (2020); Garza et al. (2023); Kadono et al. (2022); Karas et al. (2015); Kassaw et al. (2022); Walker et al. (2024); Windhagauer et al. (2021)
	*Fragilariopsis cylindrus*	Biolistics	*Sh ble*	eGFP	*FCP*	Overexpression	Gibson	Faktorova et al. (2020)
	*Pseudo‐nitzschia multistriata*	Biolistics Conjugation	*Sh ble*, *nat*	GFP	*H4*	Overexpression	Restriction enzyme Gibson	Russo et al. (2018); Sabatino et al. (2015)
	*Pseudo‐nitzschia arenysensis*	Biolistics	*Sh ble*	GFP, GUS	*H4*	Overexpression Gene silencing	Restriction enzyme	Sabatino et al. (2015, 2022)
	*Nitzschia captiva*	Biolistics	*bsr*, *Sh ble*	eGFP	*FCP*, *CfNR*	Overexpression CRISPR (failed)	Restriction enzyme	Sprecher et al. (2023)
	*Nitzschia palea*	Electroporation	*nat*	GFP	*FCP*	Overexpression	Restriction enzyme	Okada et al. (2023)
	*Nitzschia putrida*	Biolistics	*nat*	eGFP	*NADH*	Overexpression	Golden Gate	Unpublished
	*Fistulifera solaris* (*Navicula* sp.)	Biolistics Electroporation	*nptII*, *sh Ble*	GFP, eGFP	*FCP*, *H4*, *PtFCP*, *RSV*, *CaMV*	Overexpression Gene silencing	Restriction enzyme	Maeda et al. (2021); Muto et al. (2013); Naser et al. (2022)
	*Fistulifera saprophila* (*Navicula saprophila*)	Biolistics	*nptII*		*AccI*	Overexpression	Restriction enzyme	Dunahay et al. (1995)
	*Cylindrotheca fusiformis*	Biolistics	*Sh ble*	eGFP	*FCP*, *Pδ*, *CfNR*	Overexpression	Restriction enzyme	Fischer et al. (1999); Poulsen and Kroger (2005)
	*Amphora coffeaeformis*	Biolistics	*nat*	eYFP	*pPhat1*	Overexpression	Restriction enzyme	Buhmann et al. (2014)
Multipolar centrics	*Thalassiosira pseudonana*	Biolistics Electroporation Conjugation	*Sh ble*, *nat*	GFP, mEGFP YFP, mNeonGreen, mScarlet‐i, mTurquoise2	*FCP*, *U6*, *NR*, *SIT*, *Thaps3_9619*, *BST2*	Overexpression CRISPR Gene silencing	Restriction enzyme Golden Gate Gibson uLoop *In vivo*	Cochrane, Brumwell, Shrestha, et al. (2020); Falciatore et al. (2020); Hopes et al. (2016); Karas et al. (2015); Nam et al. (2022)
	*Thalassiosira weissflogii*	Biolistics		GUS	*FCP*	Overexpression	Restriction enzyme	Falciatore et al. (1999)
	*Skeletonema marinoi*	Electroporation	*bleo*		*lsu4e*, *PtFCP*	Overexpression	Restriction enzyme	Johansson et al. (2019)
	*Skeletonema costatum*	Electroporation	*bleo*		*FCP*	Gene silencing	Restriction enzyme	Zhen et al. (2024)
	*Cyclotella cryptica*	Biolistics	*nptII*, *bleo*		*AccI*, *SIT*	Overexpression Gene silencing	Restriction enzyme	Dunahay et al. (1995); Wang et al. (2023)
	*Cyclotella meneghiniana*	Conjugation	*bsr*	GUS, eGFP	*FCP*, *RL14*	Overexpression	Restriction enzyme	Yin et al. (2024)
	*Chaetoceros* sp. *CCK09*	Biolistics	*nat*	GFP	*FCP*	Overexpression	Restriction enzyme	Miyagawa‐Yamaguchi et al. (2011)
	*Chaetoceros muelleri*	Electroporation	*ble*, *nat*, *bsr*	GFP, GUS	*FCP*, *ACAT*, *U6*	Overexpression CRISPR	Restriction enzyme	Yin and Hu (2021, 2023)
	*Chaetoceros gracilis*	Electroporation	*nat*	LUC, mAG	*FCP*, *NR*	Overexpression	Restriction enzyme	Ifuku et al. (2015)

*ACAT*, acetyl‐CoA acetyltransferase; *AccI*, acetyl‐CoA carboxylase; *Act2*, purine permease, actin /actin‐like protein; *AP1*, alkaline phosphatase; *bsr*, blasticidin‐S resistance gene; BST2, bestrophin‐like protein (THAPSDRAFT_4820); *Ca1*, carbonic anhydrase; *calm*, calmodulin‐dependent protein kinase II; *CaMV*, *Cauliflower mosaic* virus 35S; *cat*, chloramphenicol acetyl transferase conferring resistance to chloramphenicol; *CfNR*, *Cylindrotheca fusiformis* nitrate reductase; *ClP*, *Chaetoceros lorenzianus*‐infecting DNA virus; *CMV*, cytomegalovirus; CRISPR, clustered regularly interspaced short palindromic repeats; DIG/pUAS, digoxin‐inducible system; *Ef2*, elongation factor 2; eGFP, enhanced green fluorescent protein gene; *Fbp1*, ferrichrome‐binding protein 1; *FCP*, fucoxanthin‐chlorophyll protein; *Flav*, flavodoxin; GFP, green fluorescent protein gene; *GLNA*, glutamine synthetase; GUS, beta‐glucuronidase; *H4*, histone H4; *Isi1*, iron‐starvation‐induced gene 1; *lsu4e*, 80S ribosomal subunit 4e; LUC, Luciferase; mAG, green fluorescence of the monomeric Azami–Green protein; mEGFP, monomeric enhanced GFP; MN, meganucleases; *NADH*, NADH–ubiquinone reductase complex 1 MLRQ subunit; *nat*, nourseothricin acetyl transferase; *nptII*, neomycin phosphotransferase II (resistance to G418/geneticin); *NR*, nitrate reductase; *Nub*, NADH:ubiquinone oxidoreductase; *oee3*, oxygen‐evolving enhancer protein 3; *Pbt*, prohibitin; PEG, polyethylene glycol (PEG)‐mediated transformation; *Phat1/ PtFCP*, *P. tricornutum* FCP promoter; *Pδ*, promoter of frua3 gene; *RL14*, ribosomal protein L14; *RSV*, *Rous sarcoma* virus long terminal repeat promoter; *RSV‐LTR*, *Rous sarcoma virus* long terminal repeat; *sat*, streptothricin acetyl transferase; *sh ble*, *Streptoalloteichus hindustanus* bleomycin resistance gene; *SIT1*, silicon transporter; *SVP*, Synaptobrevin/VAMP‐like protein; TALEN, transcription activator‐like effector nucleases; uLoop, universal Loop; *U6*, small nuclear RNA of the U6 complex; VENUS, improved version of yellow fluorescent protein; XVE/OlexA, β‐estradiol‐inducible system; YFP, yellow fluorescent protein gene; *45582*, *predicted*rotein (Phatr3_J45582).

Inducible promoters can switch gene expression on and off in response to specific environmental or experimental conditions. This allows for the controlled expression of a target gene and minimizes the potential negative effects from the overproduction of recombinant proteins that could otherwise occur during constitutive expression. The availability of nutrients can lead to significant changes in the expression of certain diatom genes. Thus, promoters that respond to nutrients, such as those involved in nitrogen, iron, silicon, and phosphate acquisition and metabolism have been used as inducible promoters. For instance, the nitrate reductase (*NR*) promoter is commonly used for inducible expression in diatoms, whereby *NR* is activated by the presence of nitrate as the sole nitrogen source and repressed if replaced by ammonium (Ifuku et al., 2015; Poulsen et al., 2006; Poulsen & Kroger, 2005; Schellenberger Costa et al., 2013). However, even when *NR* is induced, the overall transgene expression levels remain relatively low (Chu et al., 2016; Poulsen & Kroger, 2005). Three iron‐responsive promoters were functionally characterized in *P. tricornutum*, including promoters of the iron‐starvation‐induced protein1 (*Isi1*), ferrichrome‐binding protein1 (*FBP1*) and flavodoxin (*Fld*) genes (Yoshinaga et al., 2014). Promoters of a putative silicon‐related protein (Thaps3_9619) and silicon transporters (SIT) in *T. pseudonana* (*TpSIT1*, *TpSIT2*) and *C. cryptica* (*CcSIT1*) are potential alternatives because they likely have less detrimental effects on core metabolic functions compared with the *NR* promoter (Davis et al., 2017; Shrestha & Hildebrand, 2017). Likewise, the alkaline phosphatase (AP) gene is strongly induced in response to inorganic phosphate depletion (Lin et al., 2013). Testing its promoter in *P. tricornutum* (*pPhAP1*) revealed a much higher expression of transgenes compared with using either the *Fcp* or any tested *NR* promoters (Lin et al., 2017). The promoter regulating the expression of the secreted protein 1 (HASP1) gene is also responsive to phosphate depletion but repressed if phosphate is available (Slattery et al., 2022). Furthermore, there are CO_2_‐sensitive promoters available for diatom research, such as the promoter regulating the expression of the β‐carbonic anhydrase 1 gene (ptca1) in *P. tricornutum*, which is significantly decreased when cells are grown in a high CO_2_ environment (i.e., 5% CO_2_, as opposed to air‐levels of 0.04%) (Harada et al., 2005). However, it is worth mentioning that the induction and/or inactivation of certain promoters, such as the *pPhAP1*, relies on the availability of essential nutrients, which might cause transcriptional reprogramming as a response (Garza et al., 2023). Hence, the altered transcriptome might interfere with subsequent phenotyping studies to characterize the *in vivo* function of the modified target gene. To reveal if and how those associated effects imposed by the regulation of these promoters impact the physiology of the genetically modified cell lines, we suggest performing multi‐omics studies to capture potential reprogramming on diverse levels of organization from transcriptomes to metabolomes.

Heterologous promoters include non‐endogenous promoters obtained from other diatom species, non‐diatom species, viruses, and even synthetic chemical promoters. For example, the *Lhcf2* promoter from *Cylindrotheca fusiformis* was used to drive transgene expression in *P. tricornutum* and *Fistulifera* sp. (Miyagawa et al., 2009; Muto et al., 2013). With the molecular characterization of diatom‐infecting viruses (DIVs), their promoters have been studied in diverse diatoms, and some of them have shown high transgene expression (Kadono et al., 2015; Kadono et al., 2020; Kadono et al., 2022). Furthermore, promoters from the mammalian cytomegalovirus (CMV), the avian rous sarcoma virus (RSV‐LTR) and the plant cauliflower mosaic virus 35S (CaMV 35S) have been successfully used for transgene expression in *P. tricornutum* (Sakaue et al., 2008). The CaMV 35S and RSV‐LTR promoters can drive high levels of gene expression comparable to the endogenous *FcpA* promoter in the log phase of *P. tricornutum* (Kadono et al., 2020).

Furthermore, chemically inducible gene expression systems have been developed to achieve dynamic gene expression as a consequence of applying exogenous chemicals to diatom cultures. Six of these systems were tested in *P. tricornutum* (Kassaw et al., 2022). Among them, β‐estradiol and digoxin demonstrated high levels of reversibility and tunability, making them effective for the chemical induction of transgene expression. The β‐estradiol system is highly sensitive, with β‐estradiol concentrations as low as 1 nm sufficient to trigger gene expression. The digoxin‐inducible promoter enables tight control of gene expression, with activation levels directly correlating with digoxin concentrations ranging from 0 to 100 μM. Chemically inducible promoters have been used in several model organisms (e.g., *Escherichia coli* and yeast). In some cases, the use of endogenous inducible promoters carries the risk of causing unintended effects on metabolism, while constitutive promoters drive continuous expression of the target genes even when their activity is not required anymore, which can be toxic to the cell (e.g., overexpression of the Cas9 enzyme). Chemically inducible promoters overcome these limitations; however, the impact of the chemicals on the metabolism of diatoms largely is unknown and therefore needs to be empirically tested.

### Selective markers

Selective markers confer resistance to specific antibiotics/chemicals or restore auxotrophies and are often used when genetically engineering diatoms. A transgenic vector typically contains the gene(s) of interest and a selective or auxotrophic marker, the latter enabling the identification of transformants when cells are exposed to the selective agent. The selective marker genes most commonly used in diatoms confer resistance to various antibiotics, including *Shble* (phleomycin/zeocin resistance), *Nat* (nourseothricin resistance), *Bsr* (blasticidin‐S resistance), *Cat* (chloramphenicol resistance), *Sat* (streptothricin resistance) and *nptII* (G418/geneticin resistance) (Apt et al., 1996; Buck et al., 2018; Dunahay et al., 1995; Falciatore et al., 1999; Karas et al., 2015; Muto et al., 2013; Poulsen et al., 2006; Xie et al., 2014; Zaslavskaia et al., 2000). In addition, the mutation of endogenous genes can confer resistance to specific chemical compounds, which have been developed as selectable markers in diatoms. For instance, the inactivation of uridine‐5′‐monophosphate synthase (PtUMPS) and adenine phosphoribosyl transferase (PtAPT) genes in *P. tricornutum*, leading to the resistance to 5‐fluoroorotic acid (5‐FOA) and 2‐fluoroadenine (2‐FA), respectively, provides a selection strategy without relying on exogenous antibiotic resistance markers (Serif et al., 2018). Similarly, the point mutation of the endogenous phytoene desaturase gene (PDS, PHATRDRAFT_45735) conferred resistance to the herbicide norflurazon in *P. tricornutum* (Taparia et al., 2019). However, the potential effects on metabolism, following the removal of selecting agents due to the loss of endogenous genes, should be taken into consideration. Moreover, auxotrophic complementation markers provide an alternative approach. Cas9‐directed genome engineering has also been used to generate strains of *P. tricornutum* that are auxotrophic for histidine, uracil, or tryptophan; these auxotrophies can be restored by providing the respective biosynthetic gene in the transgenic vector (Slattery et al., 2020).

When considering microbial interactions involving diatoms, applying reverse genetics tools to study them is still in its infancy. Most work is still focused on managing the contamination of diatom cultures – often, the selective markers introduced during genetic engineering efforts can also be used to mitigate contamination, so long as the contaminant is susceptible to the respective antibiotic or chemical selective agent. For instance, resistance to antifungal drugs such as amphotericin, echinocandins, and azoles has been widely studied to identify key resistance genes (Jensen et al., 2015; Moirangthem et al., 2021; Morio et al., 2017; Rybak et al., 2022; Spettel et al., 2019). This work, if done with diatoms, will provide basic information for selecting antifungal markers, for instance. Furthermore, studying interactions between diatoms, bacteria, and viruses involves the depletion and rescue of bacteria and their associated viruses (Zhang et al., 2024). Thus, introducing resistance genes to diatom genomes represents an approach for controlling these interactions and therefore provides an opportunity to selectively modulate microbial communities for advancing our understanding of microbial interactions with diatoms as the host organisms.

### Reporter systems

Reporters are widely used to monitor and track engineered diatom cells by visualizing the expression of transgenes or the regulation of promoters. The integration of reporter genes allows researchers to quickly identify transformed cells and employ techniques such as fluorescence‐activated cell sorting (FACS) to isolate cells emitting fluorescence. Enzyme‐based reporters offer high sensitivity but require the addition of a substrate to generate a detectable signal (e.g., bioluminescence). For instance, luciferase (LUC) and β‐glucuronidase (GUS) genes have been expressed in several diatom species (Falciatore et al., 1999; Ifuku et al., 2015; Sabatino et al., 2015; Zaslavskaia et al., 2000). However, some reporter systems require the application of complex assays for visualization, which makes these systems more technically challenging to work with (Huttly, 2009).

**Figure 2:**
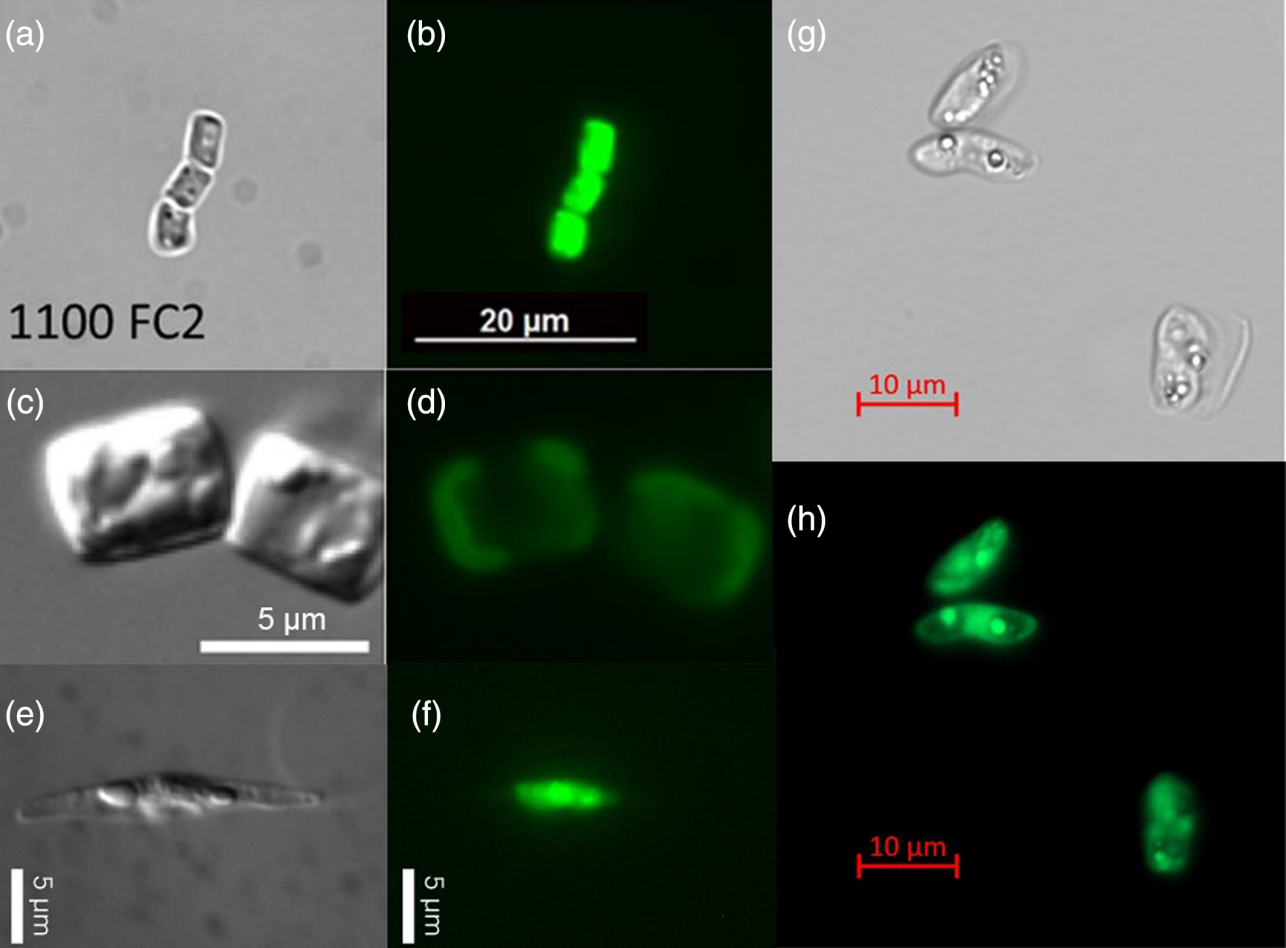
Expression of green fluorescent protein (GFP) or enhanced green fluorescent protein (eGFP) in four diatom species. Bright‐field and corresponding GFP/eGFP fluorescence images are shown for each species. (a, b) *Fragilariopsis cylindrus*, (c, d) *Thalassiosira pseudonana*, (e, f) *Phaeodactylum tricornutum*, and (g, h) *Nitzschia putrida*. Scale bars are 20 μm in (a, b), 10 μm in (g, h), and 5 μm in (c–f). Images have been taken by Jan Strauss, Amanda Hopes, and Longji Deng.

For real‐time studies and live‐cell imaging, fluorescent reporters may be better alternatives. For instance, the breakthrough of tagging fluorescent proteins revolutionized the study of protein–protein interactions in living cells, leading to the discovery of interaction networks and previously unknown protein functions (Nam et al., 2024; Turnsek et al., 2021). These proteins emit stable fluorescence upon excitation by the corresponding wavelengths of light. Because of their broad applicability (e.g., operational in different cell types, easy detectability), fluorescent proteins are perhaps the most frequently used reporter systems in diatoms (Figure 2). Examples are the green fluorescent protein (GFP), enhanced green fluorescent protein (eGFP), yellow fluorescent protein (YFP), optimized YFP (VENUS), the red fluorescent protein mCherry, and the cyan fluorescent protein gene (CFP) (Table 1). New fluorophores, including mNeonGreen, mScarlet‐i, and mTurquoise2, have been tested and validated in *T. pseudonana*, expanding the toolkit for protein localization studies in diatoms (Nam et al., 2022). Additionally, fluorescent proteins that have been established in other microalgae and diverse protists include the brightest blue fluorescent protein mTagBFP. It is believed that these fluorescent proteins should also work in diatoms (Faktorova et al., 2020). When selecting reporter genes, it is critical to consider the background conditions of the cells (e.g., presence of chlorophyll autofluorescence) to ensure the accurate detection of the reporters and to minimize interference with background fluorescence.

Various reporters offer a wide spectrum of colors, enabling researchers to select the most suitable reporter based on their objectives, including multicolor imaging to study multiple reporters simultaneously. If reporters are fused to target proteins or promoter sequences, it allows for the identification of the subcellular localization of the target genes and the regulation of the promoters under native conditions, respectively (Hao et al., 2022; Liu et al., 2016; Nojima et al., 2013; Pan et al., 2024; Shao et al., 2019). It should be noted that the folding and maturation of fluorescent reporter proteins can be a slow process that is influenced by the cellular environment (e.g., redox state). The delay between gene expression and protein maturation might introduce a bias between the detection of the fluorescence signals and the actual expression levels of the gene (Jullien & Gautier, 2015). Consequently, under such circumstances, it might be advantageous to combine the measurement of fluorescent signals with expression analyses of the target gene(s).

### Cloning and assembly strategies

Restriction enzyme cloning likely is the most common cloning method in diatom research. Both the DNA fragment of interest and the vector are cut with the same restriction enzymes at specific recognition sites. The resulting complementary sticky or blunt ends allow the DNA fragment to be ligated into the vector using a DNA ligase, creating a recombinant DNA molecule. The first application for diatoms involved inserting the *nptII* gene into a plasmid, which conferred G418 resistance to the transformed *Cyclotella cryptica* (Dunahay et al., 1995). Type II restriction enzymes have long been the workhorses in diatom reverse genetics (Poulsen et al., 2006; Sabatino et al., 2022; Wang et al., 2023).

The Gibson assembly method is based on a seamless cloning strategy that joins multiple DNA fragments together in a specific order at a constant temperature using a T5 exonuclease, a DNA polymerase, and a DNA ligase (Gibson et al., 2009). This method is based on the assembly of overlapping fragments and is not constrained by restriction enzyme sites; hence, it offers flexibility in the design of vectors. Several studies in diatom research have successfully used this assembly method (Daboussi et al., 2014; Nigishi et al., 2024; Stukenberg et al., 2018). However, it should be noted that the efficiency decreases when the number of fragments increases beyond five due to challenges with ensuring correct annealing and maintaining overlap homology between fragments (Gibson et al., 2009). Thus, nicks or any kinds of base mutations in the overhangs will significantly impact the assembly success.

The golden gate assembly uses Type IIS restriction enzymes to enable precise and scar‐free DNA assembly (Engler et al., 2008). The specified sticky ends on cuts are made at a defined distance from the recognition sites in plasmids and DNA fragments, achieving a hierarchical assembly of DNA parts (Bird et al., 2022). DNA parts are stored stably within plasmid vectors, allowing them to be efficiently reused in subsequent assembly steps. In diatoms, only *P. tricornutum* and *T. pseudonana* have been subjected to golden gate cloning (Belshaw et al., 2022; Hopes et al., 2016; Llavero‐Pasquina et al., 2022; Mooshammer et al., 2020). Some of the golden gate components for both species are available from Addgene (Watertown, MA, USA) (https://www.addgene.org/). Furthermore, the modular cloning (MoClo) system is a hierarchical and modular assembly method based on golden gate cloning, combining standardized parts, such as promoters, coding sequences, and terminators in a predefined order (https://www.addgene.org/kits/marillonnet‐moclo/). Several MoClo toolkits have been developed, providing reusable parts and vectors for mammalian (Weber et al., 2011), yeast (Lee et al., 2015), plants (Engler et al., 2014), bacteria (Iverson et al., 2016; Moore et al., 2016; Stukenberg et al., 2021), *Chlamydomonas reinhardtii* (Crozet et al., 2018), and cyanobacteria (Vasudevan et al., 2019). The use of a standardized syntax facilitates the sharing of non‐species‐specific modules (Patron et al., 2015). The MoClo toolkits have been used for diatoms (Nam et al., 2022; Russo et al., 2023) but not as commonly as other cloning methods.

The Loop assembly is another emerging and versatile DNA assembly system based on recursive DNA cloning. This method achieves high precision and reliability for assembling complex and larger constructs, with >80% average assembly efficiencies on over 200 different DNA constructs (Pollak et al., 2019). The universal Loop (uLoop) assembly, which is derived from the traditional Loop, has been successfully adapted for use in diatoms (Pollak et al., 2020). The diatom uLoop assembly kit is available from Addgene (https://www.addgene.org/kits/dupont‐diatom‐uloop/) and the uLoop library is still expanding to include new parts, such as newly characterized promoters and terminators (e.g. calm, oee3 and flav) in *P. tricornutum* (Garza et al., 2023). This open‐access diatom uLoop library provides a modular and standardized approach for constructing diverse genetic components. Thus, it fosters collaboration between research groups and ensures data reproducibility through standardization.

There is an increasing use of *in vivo* assembly, particularly for constructing larger plasmids or those requiring the integration of multiple fragments. This approach leverages *Saccharomyces cerevisiae* (i.e., yeast) protoplast transformation methods (Kouprina & Larionov, 2008). A yeast artificial chromosome (YAC), often containing a CEN‐ARS‐HIS backbone, is included on a fragment that also incorporates essential elements for replication and selection in *E. coli*. The DNA fragments used for assembly can be chemically synthesized, PCR‐amplified, or even directly obtained from isolated DNA, provided they are excised at desired positions using unique restriction enzymes. A minimum overlap of 40 base pairs (bp) between fragments is required for efficient recombination between complementary sequences; however, for assemblies involving a larger number of fragments, longer overlaps of 50–200 bp are recommended to enhance efficiency. The main advantage of this method is its capacity to assemble large plasmids, though it is more time‐consuming and demands technical expertise. Notable applications of this technique include the assembly of entire mitochondrial genomes from *P. tricornutum* and *T. pseudonana* (Cochrane, Brumwell, Shrestha, et al., 2020; Cochrane, Brumwell, Soltysiak, et al., 2020) as well as the chloroplast genome of *P. tricornutum* (Walker et al., 2023). Furthermore, recent findings have demonstrated that plasmids can be directly assembled in *P. tricornutum* through non‐homologous end joining, potentially simplifying the process of constructing genetic constructs, at least for this species (Walker et al., 2024).

## DELIVERY SYSTEMS

Microparticle bombardment has been most widely used to deliver DNA, RNA, and proteins. It has been successfully employed for both nuclear and chloroplast transformation in many microalgal species (Hopes et al., 2016; Li & Bock, 2018; Schiedlmeier et al., 1994; Sodeinde & Kindle, 1993). The DNA vectors, RNAs, or proteins are coated onto the surface of nanoparticles either made of tungsten or gold. A particle delivery system is used to deliver them into the cells under high pressure. To minimize cell damage, particles between 0.7 and 1.1 μm are usually used for diatoms (Dunahay et al., 1995). However, considering that *T. pseudonana* cells are only about three times the size of these particles, it remains to be seen what the true impact is on the integrity of the diatom cells. At least they appear to have a high regenerative potential, evidenced by restoring the original phenotypes including the elaborate silica cell walls within 2–4 weeks after bombardment (Belshaw et al., 2022; Harada et al., 2005; Moosburner et al., 2020). Despite these shortcomings (e.g., low efficiency, destructive impact on cellular integrity), microparticle bombardment has been shown to be suitable for many diatom species. DNA plasmids in the size range of 5–10 kilobases (kb) are considered suitable for biolistic manipulation (Stewart et al., 2018). Biolistics can also be used to directly deliver RNA and proteins into diatom cells (Serif et al., 2018). This approach has been effectively applied to introduce Cas9/single‐guide RNA ribonucleoprotein complexes (RNPs) for DNA‐free genome editing (Serif et al., 2018). Meanwhile, triple gene knockouts were achieved in one step by delivering six RNP complexes simultaneously, although with relatively low efficiency (15%) compared with double gene knockout (23–52%).

Electroporation introduces foreign DNA into cells by temporarily creating pores in the cell membrane through the application of a brief electric pulse (Somiari et al., 2000; Tsong, 1991). This method transfers exogenous DNA independently of the cell's abilities to take it up and has been successfully used in several diatom species (Qin et al., 2012). It shows more than 10 times higher transformation efficiency compared with conventional biolistics using small amounts of DNA (4–7 μg) (Naser et al., 2022; Yin & Hu, 2021; Zhang & Hu, 2014). Electroporation can also be used for co‐transformation using more than a single vector (Zhang & Hu, 2014). Linear plasmids can be electroporated more efficiently compared with circular plasmids, achieving up to three times greater efficiency in diatom transformation (Yin & Hu, 2021). The spheroplasting electroporation method was recently developed for *P. tricornutum* (Walker et al., 2024). This method is characterized by high efficiency in delivering episomes, with amounts of as little as 1 ng and for plasmids as large as 55.6 kb. Additionally, this method has the advantage of high survival rates of the transformed diatom cells, with growth resuming within only 2 weeks (Ifuku et al., 2015; Yin & Hu, 2021; Zhang & Hu, 2014). Electroporation is a promising strategy, but the challenge lies in optimizing electroporation conditions, including the electric field strength and pulse duration to suit different diatom species. It is assumed that the cell wall significantly impedes DNA delivery during electroporation (Azencott et al., 2007). Therefore, it is at least necessary to adjust the protocol for different diatom species, and it is likely that this method, therefore, is not as widely applicable as biolistics.

The polyethylene glycol (PEG)‐mediated transformation method is thought to facilitate the introduction of DNA into cells by promoting its passage across the cell membrane, though the precise mechanisms remain poorly understood. This method was evaluated in *P. tricornutum* during the development of the p0251s replicating plasmid (Karas et al., 2015); however, the results were highly inconsistent, yielding only a few colonies across several experiments. A recent breakthrough has occurred with the use of alcalase to protoplast *P. tricornutum* cells (Walker et al., in preparation), which dramatically increases the efficiency of PEG transformation (hundreds to thousands of transformants per reaction). This method shows great promise as it is highly efficient and does not require any specialized equipment, and it may become the preferred method for *P. tricornutum* and potentially other diatom species.

Using *E. coli* to directly introduce DNA vectors into diatom cells via bacteria‐mediated conjugation is another delivery system that was developed in the recent past (Karas et al., 2015). The donor *E. coli* strain harbors a conjugative plasmid, which encodes for the DNA transfer machinery, and an episome containing the gene(s) of interest. The episome must also contain an origin of transfer (oriT) to be mobilized during conjugation and a selective marker. Furthermore, to be stably maintained extrachromosomally, the episome must contain a suitable autonomously replicating sequence (ARS). This sequence can vary between different diatom species; without an ARS, the episome must integrate into the diatom genome for continued propagation across successive generations. This method was first developed in *P. tricornutum* and *T. pseudonana* (Karas et al., 2015) and has since been explored in other diatom species.

High‐copy number replication of the episome can be detrimental to the *E. coli* donor strain, particularly when CRISPR/Cas9 systems or other deleterious genes are expressed. To address the unstable characteristics of high‐copy number plasmids, low‐copy number and medium‐copy number episomes have been developed. The pCC1BAC backbone (present in p0251s, Karas et al., 2015) contains two origins of replication: one that is constitutively expressed and facilitates single‐copy number replication, and another that can be induced to high‐copy number replication in the presence of L‐arabinose when maintained in the EPI300 *E. coli* strain. Inducing high‐copy number expression may be deleterious to the cell, but it can be useful when isolating large quantities of the episome for sequencing or other downstream applications. Another diatom episome, pPtPBR1, allows for constitutive medium‐copy number expression in *E. coli* (Diner et al., 2016).

When using bacterial conjugation to deliver CRISPR/Cas9‐containing episomes, this system achieves a comparable percentage of biallelic mutations when compared with microparticle bombardment (Moosburner et al., 2020; Sharma et al., 2018; Slattery et al., 2018). Compared with genome‐integrated DNA vectors containing the Cas9 gene, extrachromosomal episomes carrying CRISPR/Cas9 systems can be easily removed by growing the diatom cells under non‐selective conditions. This causes the loss of the episome over time (i.e, episome curing), which therefore avoids unwanted mutations by the constitutive expression of Cas9 if this gene has become part of the diatom genome. Thus, bacterial conjugation has the advantage of delivering genetic constructs without altering the host genome. However, the assembly of plasmids targeting multiple genes can lead to the instability of the constructs in *E. coli*, which needs to be considered when designing more complex multi‐target constructs (Taparia et al., 2021).

## CELL‐LINE SCREENING POST‐TRANSFORMATION

To isolate and confirm the desired genetic modifications, steps include subcloning and screening. Both are necessary because the initial cultures are often mosaic (Huang & Daboussi, 2017; Serif et al., 2018; Weyman et al., 2015), necessitating the isolation of single‐cell lines on solid plates or in liquid medium. Most transformed diatom colonies can be isolated using antibiotics or chemical compounds on selective plates. However, the growth conditions on these plates should be tested when working with a novel diatom species. Clonal cell lines generally appear after the first round of subcloning. However, mono‐allelic (heterozygous) mutations frequently occur likely due to incomplete editing. In such cases, a second round of subcloning is required to obtain bi‐allelic (homozygous) mutations in the targeted gene. However, this process is time‐consuming, taking more than 1 week to obtain clones, depending on the growth rates and the delivery system used (Belshaw et al., 2022; Yin & Hu, 2021). Transformed cells expressing a fluorescence marker can be separated by fluorescence‐activated cell sorting (FACS), which therefore can achieve high rates of cell recovery under near axenic conditions (Nunez, 2001; Pereira et al., 2018). However, it should be noted that some diatom species may not survive the FACS procedure due to mechanical pressure, which may cause disruptions of the plasma membrane and cell wall (Reckermann, 2000).

PCR amplification of the target gene followed by analyzing the electropherograms is a common method for analyzing the genetic mutation to be expected (Hopes et al., 2016; Moosburner et al., 2022; Nymark et al., 2016). For gene overexpression and knockdown, selective marker or reporter genes are typically amplified for initial screening (De Riso et al., 2009; Haslam et al., 2020). Direct amplification of the target gene confirms its presence if the overexpressed gene is exogenous (Strauss et al., 2023). Gene‐knockout cells often exhibit a distinct PCR‐based genotyping pattern compared to WT cells (Hopes et al., 2016). When a small deletion occurs (less than 100 bp or a few hundred bp), gene‐knockout cell lines typically display a smaller band than WT due to the reduced fragment size. Conversely, if the deletion spans several kilobases, no amplification is observed, necessitating additional confirmation using primers targeting the 3′ and 5′ flanking regions outside the deleted area (Belshaw et al., 2022). To further validate the modification, the expected sequence is cloned into a vector for transformation into *E. coli*, followed by Sanger sequencing and alignment with the reference genome. Additionally, alternative methods such as high‐resolution melt curve analysis (HRM), T7 endonuclease I assay (T7EI), and software‐based Tracking of Indels by Deconvolution (TIDE) provide insight into the nature of genetic modifications (Moosburner et al., 2020; Moosburner et al., 2022; Nymark et al., 2016; Slattery et al., 2018). If PCR reactions fail due to extensive genomic rearrangements, Southern blotting may be useful to detect fragment size shifts and confirm copy number variations compared to WT DNA (Kira et al., 2016; Zhang & Hu, 2014). To further corroborate the genetic modifications of coding genes, relative protein levels can be assessed by Western blotting, whereas mRNA levels of the target gene can be analyzed using real‐time quantitative PCR (RT‐qPCR) (Gorlich et al., 2019; Strauss et al., 2023).

## PROSPECTS

Genetic engineering in diatoms has come a long way, and there are significant prospects because of three reasons: (i) Currently, there is a step change in the development of diatom genomics and multi‐omics resources, which provide the foundation for discovering novel biology and the application of genetic tools to new species. (ii) Diatoms are as genetically tractable as any established model organisms in biology, that is, *Arabidopsis thaliana*, *yeast*, *Caenorhabditis elegans*. However, diatoms, unlike those established biological models, represent globally relevant organisms underpinning the largest food webs on Earth, and they drive global biogeochemical cycles responsible for the habitability of our planet. (iii) Their fast growth, high content of lipids, essential fatty acids, and their elaborate nanopatterned silica cell walls make them a target of the biotechnology sector, from nutraceuticals to material science. Consequently, for human societies to benefit from diatoms, we will need to continue to develop their genetic tractability. One current frontier is to design the first synthetic diatom. To achieve that goal, synthetic chromosomes need to be assembled, transformed, and expressed in a diatom host, which remains challenging, but current work in this exciting field of research paves the way for building the first synthetic diatom soon. With respect to the latter, entire ~500 kbp chromosomes of *P. tricornutum* were successfully assembled in yeast and subsequently transferred to *E. coli*, establishing a critical proof of concept that demonstrates the ability of these organisms to maintain large diatom DNA fragments (Karas et al., 2013). Building on these results, it is proposed that all 25 chromosomes of *P. tricornutum* could be redesigned and resynthesized as 50 chromosomes that are ~400–500 kbp in size (Pampuch et al., 2022). This approach would allow for assembly in yeast and efficient propagation in *E. coli*. Furthermore, a recently developed rapid method for chloroplast genome assembly (Walker & Karas, 2025) could accelerate the creation of synthetic genomes with enhanced features, such as the removal of non‐essential elements (e.g., repetitive sequences, transposons), genome recoding, and architectural reorganization. The delivery of whole chromosomes is anticipated to be possible through bacterial conjugation. Additionally, smaller fragments of approximately 50 kbp can now be delivered using an optimized electroporation protocol, enabling parallel testing of synthetic constructs (Walker et al., 2024). These recent advancements in the field of diatom genetic engineering, combined with many high‐resolution reference genomes that will become available soon, position diatoms as robust and promising organisms for synthetic genomics to advance fundamental biological research and biotechnology (Boxes 1 and 2).

Box 1 **Summary**

- Diatoms are important primary producers, and they are being used for diverse biotechnological applications.
- Multiomics resources available for many diatom species facilitate genetic engineering.
- Molecular tool kits include diverse promoters, selectable markers, and reporter systems.
- Cloning and assembling strategies have been developed even for large plasmids.
- The latest reverse genetics tools aim at building the first synthetic diatom.

Box 2 **Open questions**

- Considering that diatoms are the most species‐rich group of algae, how many diatom species do we need to develop into model systems to address the most significant fundamental and applied questions?
- What kind of genetic engineering is required to identify the mechanisms underpinning the interactions of diatoms with other species?
- How can genetic engineering help to make use of diatoms in fast‐emerging fields such as energy technology?
- Will it be possible to generate the first synthetic diatom for the development of novel carbon‐capture mechanisms and the sustainable synthesis of high‐value products?

## AUTHOR CONTRIBUTIONS

YL conceived and designed the structure of this manuscript, and she wrote the first version. All authors contributed to writing and editing the manuscript. LD designed Figures 1 and 2.

## CONFLICT OF INTEREST

The authors declare no conflict of interest.

## Supporting information


**Table S1.** Summary of sequenced diatom genomes. Resource from National Center for Biotechnology Information (NCBI), Joint Genome Institute (JGI), European Nucleotide Archive (ENA), Ensembl genome browser, DiatOmicBase, and PLAZA.

## Data Availability

The data of this study are available as part of the main article and the supplementary material.
