# Supplementary material for: Genetic engineering in diatoms: advances and prospects
Source: Plant J. 2025 Mar 16;121(6):e70102. doi: 10.1111/tpj.70102 (PMC11910954; doi:10.1111/tpj.70102)
Supplement: Supplementary file 1 — Table S1. Summary of sequenced diatom genomes. Resource from National Center for Biotechnology Information (NCBI), Joint Genome Institute (JGI), European Nucleotide Archive (ENA), Ensembl genome browser, DiatOmicBase, and PLAZA. [file TPJ-121-0-s001.docx]

**Table S1.** Summary of sequenced diatom genomes. Resource from National Center for Biotechnology Information (NCBI), Joint Genome Institute (JGI), European Nucleotide Archive (ENA), Ensembl genome browser, DiatOmicBase, and PLAZA.

| **Species** | | **Strain** | **Link** | **Resource** |
| --- | --- | --- | --- | --- |
| Raphic pennate | *Phaeodactylum tricornutum* | CCAP 1055/1 | https://phycocosm.jgi.doe.gov/Phatr2_bd/Phatr2_bd.home.html | JGI |
|  |  |  | https://www.ncbi.nlm.nih.gov/datasets/genome/GCF_000150955.2/ | NCBI |
|  |  |  | https://protists.ensembl.org/Phaeodactylum_tricornutum/Info/Index/ | Ensembl |
|  |  |  | https://bioinformatics.psb.ugent.be/plaza/versions/plaza_diatoms_01/organism/view/Phaeodactylum+tricornutum | Plaza |
|  | *Mayamaea pseudoterrestris* | NIES-4280 | https://phycocosm.jgi.doe.gov/Maypse1/Maypse1.home.html | JGI |
|  |  |  | https://www.ncbi.nlm.nih.gov/datasets/genome/GCA_027923505.1/ | NCBI |
|  | *Fistulifera solaris* | JPCC DA0580 | https://www.ncbi.nlm.nih.gov/datasets/genome/GCA_002217885.1/ | NCBI |
|  |  |  | https://phycocosm.jgi.doe.gov/Fisso2/Fisso2.home.html | JGI |
|  |  |  | https://bioinformatics.psb.ugent.be/plaza/versions/plaza_diatoms_01/organism/view/Fistulifera+solaris | Plaza |
|  | *Fistulifera pelliculosa* | UTEX661 | https://www.ncbi.nlm.nih.gov/datasets/genome/GCA_019693425.1/ | NCBI |
|  |  | CCMP 543 | https://www.ncbi.nlm.nih.gov/datasets/genome/GCA_026008555.1/ | NCBI |
|  | *Amphora coffeaeformis* | CCMP126 | https://www.ncbi.nlm.nih.gov/sra/SRP046053 | NCBI |
|  | *Epithemia pelagica.* | UHM3201 | https://www.ebi.ac.uk/ena/browser/view/PRJEB54946 | ENA |
|  | *Epithemia catenata* |  | https://www.ncbi.nlm.nih.gov/datasets/genome/GCA_964019935.1/ | NCBI |
|  | *Nitzschia inconspicua* | GAI-293 | https://phycocosm.jgi.doe.gov/Nithil2/Nithil2.home.html | JGI |
|  |  |  | https://www.ncbi.nlm.nih.gov/datasets/genome/GCA_019154785.2/ | NCBI |
|  |  |  | https://www.ebi.ac.uk/ena/browser/view/GCA_019154785.2 | ENA |
|  | *Seminavis robusta* | D6 | https://bioinformatics.psb.ugent.be/plaza/versions/plaza_diatoms_01/organism/view/Seminavis+robusta | Plaza |
|  |  |  | https://phycocosm.jgi.doe.gov/Semro1_1/Semro1_1.home.html | JGI |
|  |  |  | https://www.ncbi.nlm.nih.gov/datasets/genome/GCA_903772945.1/ | NCBI |
|  | *Nitzschia palea* | CPCC-160 | https://www.ncbi.nlm.nih.gov/datasets/genome/GCA_019593585.1/ | NCBI |
|  | *Cylindrotheca fusiformis* | UTEX2084 | https://www.ncbi.nlm.nih.gov/datasets/genome/GCA_019693525.1/ | NCBI |
|  | *Cylindrotheca Closterium* |  | https://phycocosm.jgi.doe.gov/Cylclo1/Cylclo1.home.html | JGI |
|  |  |  | https://www.ncbi.nlm.nih.gov/datasets/genome/GCA_933822405.4/ | NCBI |
|  | *Fragilariopsis kerguelensis* |  | https://www.ncbi.nlm.nih.gov/datasets/genome/GCA_902825245.1/ | NCBI |
|  | *Fragilariopsis cylindrus* | CCMP1102 | https://www.diatomicsbase.bio.ens.psl.eu/genomeBrowser?species=Phaeodactylum+tricornutum | DiatOmicBase |
|  |  |  | https://mycocosm.jgi.doe.gov/Fracy1/Fracy1.home.html | JGI |
|  |  |  | https://protists.ensembl.org/Fragilariopsis_cylindrus_ccmp1102_gca_001750085/Info/Index | Ensembl |
|  |  |  | https://www.ncbi.nlm.nih.gov/datasets/genome/GCA_001750085.1/ | NCBI |
|  | *Pseudo-nitzschia multistriatab* | B856 | https://www.diatomicsbase.bio.ens.psl.eu/genomeBrowser?species=Pseudo-nitzschia+multistriata | DiatOmicBase |
|  |  |  | https://www.ncbi.nlm.nih.gov/datasets/genome/GCA_900660405.1/ | NCBI |
|  |  |  | https://protists.ensembl.org/Pseudonitzschia_multistriata/Info/Index | Ensembl |
|  | *Pseudo-nitzschia arenysensis* | MMETSP0329 | https://github.com/johnsolk/MMETSP | MMETSP-github |
|  |  |  | https://www.ebi.ac.uk/ena/browser/view/GKNO01000000?show=sample-attributes | ENA |
|  | *Pseudo-nitzschia delicatissima* | CNS00130 | https://www.ncbi.nlm.nih.gov/datasets/genome/GCA_037355735.1/ | NCBI |
|  | *Pseudo-nitzschia multiseries* | CLN-47 | https://mycocosm.jgi.doe.gov/Psemu1/Psemu1.home.html | JGI |
|  |  |  | https://www.ncbi.nlm.nih.gov/datasets/genome/GCA_037355745.1/ | NCBI CNS00149 |
|  |  |  | https://bioinformatics.psb.ugent.be/plaza/versions/plaza_diatoms_01/organism/view/Pseudo-nitzschia+multiseries | Plaza |
|  | *Pseudo-nitzschia pungen* | CNS00055 | https://www.ncbi.nlm.nih.gov/datasets/genome/GCA_037355855.1/ | NCBI |
|  | *Nitzschia putrida* | NIES-4239 | https://www.ncbi.nlm.nih.gov/datasets/genome/GCA_016586335.1/ | NCBI |
|  |  |  | https://mycocosm.jgi.doe.gov/Nitput1/Nitput1.home.html | JGI |
| Araphic pennates | *Asterionellopsis glacialis* | A3 | https://www.ncbi.nlm.nih.gov/datasets/genome/GCA_014885115.2/ | NCBI |
|  | *Psammoneis japonica* | ECT2AJA-110 | https://www.ncbi.nlm.nih.gov/datasets/genome/GCA_008632985.1/ | NCBI |
|  | *Asterionella formosa* | BG1 | https://www.ncbi.nlm.nih.gov/datasets/genome/GCA_002256025.1/ | NCBI |
|  | *Licmophora abbreviata* | CCMP470 | https://www.ncbi.nlm.nih.gov/datasets/genome/GCA_900291995.1/ | NCBI |
|  | *Fragilaria crotonensis* | SAG 28.96 | https://www.ncbi.nlm.nih.gov/datasets/genome/GCA_022925895.1/ | NCBI |
| multipolar centrics | *Thalassiosira oceanica* | CCMP1005 | https://mycocosm.jgi.doe.gov/Thaoce1/Thaoce1.home.html | JGI |
|  |  |  | https://protists.ensembl.org/Thalassiosira_oceanica_gca_000296195/Info/Index | Ensembl |
|  |  |  | https://bioinformatics.psb.ugent.be/plaza/versions/plaza_diatoms_01/organism/view/Thalassiosira+oceanica | Plaza |
|  |  |  | https://www.ncbi.nlm.nih.gov/datasets/genome/GCA_000296195.2/ | NCBI |
|  | *Skeletonema potamos* | AJA081-3 | https://www.ncbi.nlm.nih.gov/datasets/genome/GCA_036940105.1/ | NCBI |
|  | *Skeletonema marinoi* | CNS00100 | https://www.ncbi.nlm.nih.gov/datasets/genome/GCA_030544225.1/ | NCBI |
|  | *Skeletonema dohrnii* | AJA304-46 | https://www.ncbi.nlm.nih.gov/datasets/genome/GCA_036939515.1/ | NCBI |
|  | *Skeletonema tropicum* | CNS00166 | https://www.ncbi.nlm.nih.gov/datasets/genome/GCA_037178625.1/ | NCBI |
|  | *Skeletonema grethae* | AJA274-08 | https://www.ncbi.nlm.nih.gov/datasets/genome/GCA_036939775.1/ | NCBI |
|  | *Skeletonema costatum* | RCC75 | https://www.ncbi.nlm.nih.gov/datasets/genome/GCA_018806925.1/ | NCBI |
|  | *Minidiscus variabilis* | CCMP495 | https://phycocosm.jgi.doe.gov/Mintr2/Mintr2.home.html | JGI |
|  | *Thalassiosira hispida* | AJA318-38 | https://www.ncbi.nlm.nih.gov/datasets/genome/GCA_036939495.1/ | NCBI |
|  | *Thalassiosira allenii* | AJA297-05 | https://www.ncbi.nlm.nih.gov/datasets/genome/GCA_036939655.1/ | NCBI |
|  | *Thalassiosira profunda* | ECT2AJA-044 | https://www.ncbi.nlm.nih.gov/datasets/genome/GCA_036939355.1/ | NCBI |
|  | *Detonula confervacea* | CCMP975 | https://www.ncbi.nlm.nih.gov/datasets/genome/GCA_036939415.1/ | NCBI |
|  |  | CCMP995 | https://www.ncbi.nlm.nih.gov/datasets/genome/GCA_036939375.1/ | NCBI |
|  | *Shionodiscus oestripii* | AJA294-31 | https://www.ncbi.nlm.nih.gov/datasets/genome/GCA_036939715.1/ | NCBI |
|  | *Thalassiosira tumida* | CCMP1469 | https://www.ncbi.nlm.nih.gov/datasets/genome/GCA_037356255.1/ | NCBI |
|  | *Thalassiosira gravida* | AJA262-17 | https://www.ncbi.nlm.nih.gov/datasets/genome/GCA_037356215.1/ | NCBI |
|  | *Thalassiosira delicatula* | AJA262-20 | https://www.ncbi.nlm.nih.gov/datasets/genome/GCA_036939835.1/ | NCBI |
|  |  | RCC2560 | https://www.ncbi.nlm.nih.gov/datasets/genome/GCA_036939285.1/ | NCBI |
|  | *Thalassiosira pacifica* | AJA261-08 | https://www.ncbi.nlm.nih.gov/datasets/genome/GCA_036939875.1/ | NCBI |
|  | *Discostella stelligera* | AJA275-18 | https://www.ncbi.nlm.nih.gov/datasets/genome/GCA_036939735.1/ | NCBI |
|  | *Discostella pseudostelligera* | AJA075-4 | https://www.ncbi.nlm.nih.gov/datasets/genome/GCA_036940085.1/ | NCBI |
|  |  | AJA232-27 | https://www.ncbi.nlm.nih.gov/datasets/genome/GCA_036939915.2/ | NCBI |
|  | *Stephanodiscus minutulus* | AJA356-17 | https://www.ncbi.nlm.nih.gov/datasets/genome/GCA_036939435.1/ | NCBI |
|  | *Cyclostephanos tholiformis* | AJA228-03 | https://www.ncbi.nlm.nih.gov/datasets/genome/GCA_036939975.2/ | NCBI |
|  | *Cyclostephanos invisitatus* | AJA276-04 | https://www.ncbi.nlm.nih.gov/datasets/genome/GCA_036939675.1/ | NCBI |
|  | *Thalassiosira pseudonana* | CCMP1335 | https://www.ncbi.nlm.nih.gov/datasets/genome/GCF_000149405.2/ | NCBI |
|  |  | CCMP1335 | https://mycocosm.jgi.doe.gov/Thaps3/Thaps3.home.html | NCBI |
|  |  | CCMP1335 | https://protists.ensembl.org/Thalassiosira_pseudonana/Info/Index?db=core | Ensembl |
|  |  | CCMP1335 | https://bioinformatics.psb.ugent.be/plaza/versions/plaza_diatoms_01/organism/view/Thalassiosira+pseudonana | Plaza |
|  |  | CCMP1335 | https://www.diatomicsbase.bio.ens.psl.eu/genomeBrowser?species=Thalassiosira+pseudonana | DiatOmicBase |
|  | *Cyclotella distinguenda* | AJA349-1 | https://www.ncbi.nlm.nih.gov/datasets/genome/GCA_036939455.1/ | NCBI |
|  | *Cyclotella choctawhatcheeana* | AJA259-04 | https://www.ncbi.nlm.nih.gov/datasets/genome/GCA_036939855.1/ | NCBI |
|  | *Cyclotella atomus* | AJA010-31 | https://www.ncbi.nlm.nih.gov/datasets/genome/GCA_036940135.2/ | NCBI |
|  |  | AJA232-09 | https://www.ncbi.nlm.nih.gov/datasets/genome/GCA_036939935.1/ | NCBI |
|  |  | WR299-11 | https://www.ncbi.nlm.nih.gov/datasets/genome/GCA_036939575.1/ | NCBI |
|  | *Cyclotella cryptica* | CCMP332 | https://phycocosm.jgi.doe.gov/Cyccr2_2/Cyccr2_2.home.html | NCBI |
|  |  | CCMP332 | https://www.ncbi.nlm.nih.gov/datasets/genome/GCA_013187285.2/ | NCBI |
|  |  | AJA263-27 | https://www.ncbi.nlm.nih.gov/datasets/genome/GCA_036939815.1/ | NCBI |
|  | *Cyclotella cf. meneghiniana* | AJA164-11 | https://www.ncbi.nlm.nih.gov/datasets/genome/GCA_036940065.1/ | NCBI |
|  | *Cyclotella kingstonii* | AJA303-46 | https://www.ncbi.nlm.nih.gov/datasets/genome/GCA_036939535.1/ | NCBI |
|  | *Conticribra guillardii* | CCMP988 | https://www.ncbi.nlm.nih.gov/datasets/genome/GCA_036939335.1/ | NCBI |
|  | *Conticribra weissflogii* | AJA158-1 | https://www.ncbi.nlm.nih.gov/datasets/genome/GCA_036940025.1/ | NCBI |
|  |  | CCMP 1336 | https://phycocosm.jgi.doe.gov/Conwei1/Conwei1.info.html | JGI |
|  | *Porosira glacialis* | RCC2039 | https://www.ncbi.nlm.nih.gov/datasets/genome/GCA_036939395.1/ | NCBI |
|  | *Skeletonema menzelii* | AJA215-34 | https://www.ncbi.nlm.nih.gov/datasets/genome/GCA_036940005.1/ | NCBI |
|  | *Chaetoceros gracilis* | UTEX LB 2658 | https://chaetoceros.nibb.ac.jp/ | ChaetoBase |
|  | *Chaetoceros tenuissimus* | NIES-3715 | https://www.ncbi.nlm.nih.gov/datasets/genome/GCA_021927905.1/ | NCBI |
|  | *Chaetoceros muellerii* | NMCA1316 | https://www.ncbi.nlm.nih.gov/datasets/genome/GCA_019693545.1/ | NCBI |
